# Sex differences in number of X chromosomes and X-chromosome inactivation in females promote greater variability in hearing among males

**DOI:** 10.1186/s13293-022-00457-9

**Published:** 2022-09-16

**Authors:** Van Summers

**Affiliations:** Independent Researcher, Laurel, MD USA

**Keywords:** Sex differences, Greater male variability, X-chromosome inactivation, Hearing

## Abstract

**Background:**

For more than 150 years, research studies have documented greater variability across males than across females (“greater male variability”—GMV) over a broad range of behavioral and morphological measures. In placental mammals, an ancient difference between males and females that may make an important contribution to GMV is the different pattern of activation of X chromosomes across cells in females (mosaic inactivation of one the two X chromosomes across cells) vs males (consistent activation of a single X chromosome in all cells). In the current study, variability in hearing thresholds was examined for human listeners with thresholds within the normal range. Initial analyses compared variability in thresholds across males vs. across females. If greater across-male than across-female variability was present, and if these differences in variability related to the different patterns X-chromosome activation in males vs. females, it was expected that correlations between related measures *within* a given subject (e.g., hearing thresholds at given frequency in the two ears) would be greater in males than females.

**Methods:**

Hearing thresholds at audiometric test frequencies (500–6000 or 500–8000 Hz) were extracted from two datasets representing more than 8500 listeners with normal hearing (4590 males, 4376 females). Separate data analyses were carried out on each dataset to compare: (1) relative variability in hearing thresholds across males vs. across females at each test frequency; (2) correlations between both across-ear and within-ear hearing thresholds within  males vs. within  females, and (3) mean thresholds for females vs. males at each frequency.

**Results:**

A consistent pattern of GMV in hearing thresholds was seen across frequencies in both datasets. In addition, both across-ear and within-ear correlations between thresholds were consistently greater in males than females. Previous studies have frequently reported lower mean thresholds for females than males for listeners with normal hearing. One of the datasets replicated this result, showing a clear and consistent pattern of lower mean thresholds for females. The second data set did not show clear evidence of this female advantage.

**Conclusions:**

Hearing thresholds showed clear evidence of greater variability across males than across females and higher correlations across related threshold measures within males than within females. The results support a link between the observed GMV and the mosaic pattern of X-activation for females that is not present in males.

**Supplementary Information:**

The online version contains supplementary material available at 10.1186/s13293-022-00457-9.

## Highlights

- Greater variability in hearing thresholds across males than females for human listeners with thresholds within the normal range.
- Higher within-ear and between-ear correlations between thresholds for males than females consistent with sex chromosome effects on variability
- NIH-mandated inclusion of sex as a biological variable should include sex differences in variability and underlying mechanisms

**Supplementary Information:**

The online version contains supplementary material available at 10.1186/s13293-022-00457-9.

## Introduction

Dating back at least as far as Charles Darwin, scientists have discussed the “greater male variability” (GMV) seen in many species, with males tending to show more variability than females on a range of behavioral and morphological measures [1–5]. Most of the research on GMV has focused on humans and specifically on human brains and cognitive abilities. However, research on other species and other phenotypic properties indicates that GMV is not limited to humans [6, 7] or brains [4]. In addition, GMV has been identified across the lifespan beginning at birth, suggesting that genetic and possibly in utero developmental factors may interact to play an important role in these sex-linked differences. Measures showing evidence of GMV across the lifespan include body weight (at birth and in adults), blood parameters, and a range of measures of brain structure [4, 8, 9]. An improved understanding of the factors underlying the GMV seen in many human characteristics should benefit our understanding of sex differences in vulnerability to disease and in a range of additional phenotypic traits and anatomic characteristics.

Evolutionary mechanisms associated with natural and sexual selection have been posited as contributing to or accounting for GMV [1, 6, 10–12]. However, a mechanism that predates extant mammalian species by more than 100 million years may make an important contribution to GMV in phenotypic traits of placental mammals. That mechanism is the different patterns of X-chromosome activation across cells of females vs. males [7, 13, 14].

**Fig. 1:**
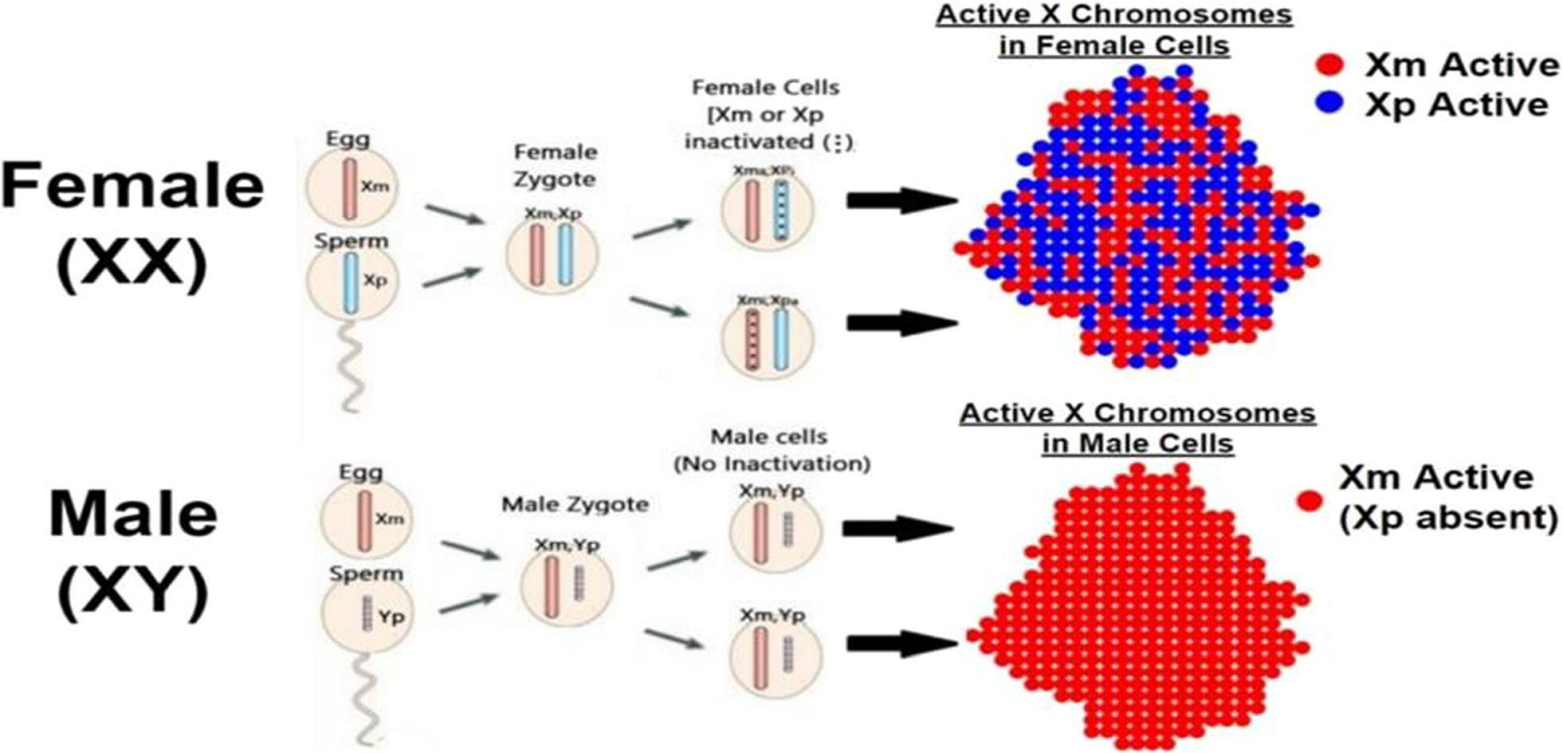
Schematic representation of X-chromosome activation in males vs. females. In placental mammals, females show a mosaic pattern of activation with one of the two X chromosomes activated in each cell. Males show consistent activation of the single (maternally contributed) X chromosome across all cells

In placental mammals, the sex chromosomes are heterogametic (XY) for males and homogametic (XX) for females. The Y chromosome contains a very limited number of genes including the SRY gene that provides instructions for the development of male gonads. The X chromosome, on the other hand, contains over 1000 genes influencing many phenotypic properties [15]. For males, the single X chromosome is activated in every cell throughout the body. For females, very early in prenatal development, each cell of the embryo inactivates one of its two X chromosomes, at random, and all subsequent daughter cells follow the “decision” made by their progenitor cell. The purpose of this inactivation is “dosage compensation” [16]. Because males have only one X chromosome, every gene on that chromosome must be fully capable of producing the effects it is designed for, and if both X chromosomes were functional in females, they would receive a “double dose”, which could be problematic if not lethal. Accordingly, females inactivate one of the X chromosomes in every cell of their bodies. One result of the early, random inactivation of one or the other X chromosome is that females exhibit mosaic patterns of X-gene expression across their bodies but males do not (see Fig. 1). This male–female difference is an attractive candidate as possibly contributing to GMV given that, like GMV, it is present across eutherian species, across anatomical regions, and is present early in development (in utero).

For a range of X-linked syndromes and diseases, GMV is the result of more males being in the negative tails of distributions. Specifically, males are affected more severely than females in more than 500 X-linked diseases [17]. A considerable amount of research has focused on this tail of various distributions when discussing GMV [17–19]. If GMV influenced only the negative tail of a given distribution, the mean of the male distribution always should be shifted lower than for females. However, in many instances, GMV on a given trait is present without clear differences in the means. In still other cases, GMV is paired with higher mean values for males than females. Overall there does not appear to be any consistent association between sex-related differences in variability and in mean scores [19, 20]. For many traits and morphological measures, GMV is characterized by more males being present in both the positive and negative tails of distributions that are flatter than those for females [6, 21].

**Fig. 2:**
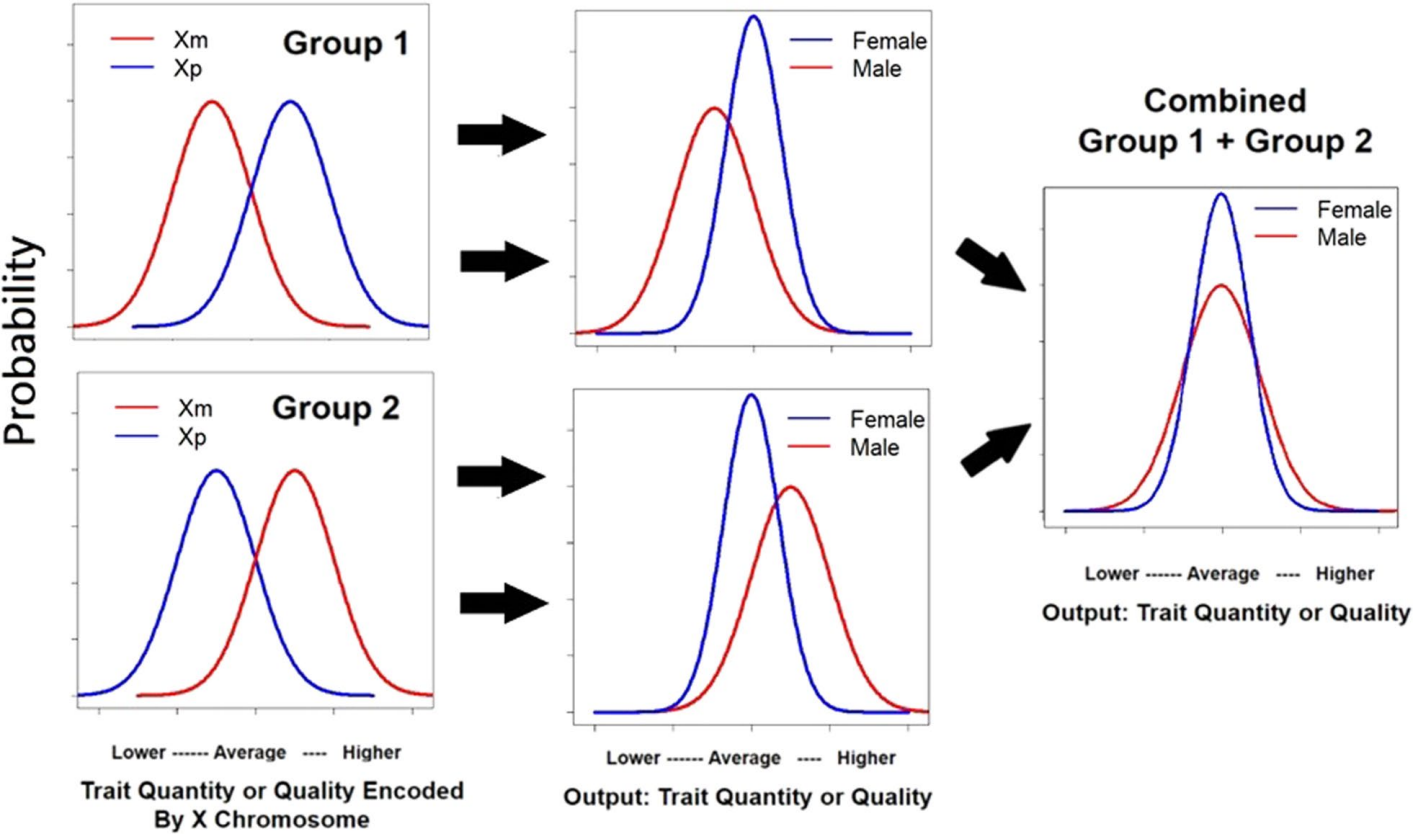
Combined influence of two X chromosomes can reduce phenotypic variability in females relative to males. For X-linked traits, the combined influence of two X chromosomes in females vs. the influence of a single X chromosome in males can produce GMV in trait quantity or quality (see text)

One way of accounting for more males in both the positive and negative tails of the distribution for a given trait is to posit separate mechanisms for the two tails. For example, increased vulnerability to X-linked diseases in males, based on an adverse mutation on their single X chromosome, will place more males in the negative tails for these diseases, while sexual selection by females of males with extreme variants of various traits may place more males in the positive tails of these distributions [11, 22, 23]. However, a single mechanism, the mosaic pattern of X-inactivation of two X chromosomes in females and the activation of a single X chromosome in males, may lead to more values in both tails of male distributions. Figure 2 illustrates one way this could occur. A starting assumption in this account is that the quantity or quality of some trait is coded by genes on X chromosomes of the parents of a son or daughter. The left hand panels of the figure represent the quantity or quality of this trait based on the contribution of the X chromosome from each parent. The upper panels represent Group 1 where the X chromosome contributed by the father (Xpaternal = Xp) encodes a higher quantity or quality for the trait than the X chromosome from the mother (Xmaternal = Xm). For daughters, the resulting quantity or quality on the trait is based on an averaging of these two distributions—represented by the curve for females in the upper middle panel of the figure. The curve for males in this panel is based only on the contribution from Xm and is lower than the curve for females. The lower panels represent Group 2 where the situation is reversed and Xm encodes a higher quantity or quality on the trait than Xp. For females, quantity or quality on the trait is again based on an averaging of the two curves so the curve for females in the lower middle panel matches the same curve in the panel above it. However, for Group 2, males are at an advantage relative to the females since their curve is entirely based on Xm which contains alleles that encode a higher quantity of or quality on the trait than Xp. The right-hand panel of the figure combines the distributions for Group 1 and Group 2. In these combined distributions, the distribution for males is flatter with more values in each tail.

It should be noted that the averaging of the Xm and Xp distributions to produce the distributions for females in the center panels of Fig. 2 weighted the Xm and Xp distributions equally. This is appropriate when random selection of Xm or Xp for inactivation produces approximately equal contributions from Xm and Xp across cells. This may occur in about 50% of the female population [24]. Imbalanced (skewed) patterns of activation, resulting in greater contributions from either Xm or Xp, are also common in many females. However, this imbalance rarely approaches 100%, unlike males where it is always 100%. A study examining X-inactivation patterns in blood samples from 1005 females reported that only 8% showed imbalances of 80% or more [25]. Based on the current account, the subset of females showing strong imbalance in the expression of Xm vs. Xp would be expected to show greater variability, more similar to variability in males, than females as a whole.^1^

For listeners with hearing thresholds within the normal range, females often show a small advantage in absolute hearing sensitivity, detecting slightly lower-amplitude tones at threshold [26–28]. If these differences reflect a benefit from a mosaic pattern of activation of two X chromosomes for tone detection, and if the absence of mosaic activation in males is linked to GMV, then tone detection thresholds should show greater variability in males than females, even among listeners with normal hearing. We report hearing data below that examine this hypothesis.

Visual comparison of the mosaic vs. uniform X-activation patterns for females vs. males in Fig. 1 might lead to an expectation of greater female than male variability due to the greater variability in the X-activation pattern within females than within males. Note, however, that this greater female variability is within an individual female relative to an individual male, rather than variability between males relative to variability between females. The variable (mosaic) pattern of X-activation within a given female vs. the uniform pattern in a given male leads to the prediction that correlations among different, related measures should be higher for males than females. These higher correlations in males re: females have been reported for various anatomical measures across brain regions and have been linked to influences of mosaic X-activation in females on these correlations [6, 8, 9, 21]. Greater correlations across related measures in males than in females (greater male correlations—GMCs) and GMV across subjects each may suggest an X-linked influence on a given behavioral or morphological characteristic.

Auditory hair cells in the cochlea transduce displacements of cochlear fluid into electrochemical neural signals that are then propagated to higher auditory centers in the brain. Wu et al. [29] reported that in female mice, these auditory hair cells show a mosaic pattern characterized by “fine-grained intermingling” of hair cells with either the maternally or paternally contributed X chromosome activated. These findings make auditory processing a good place to look for the proposed links between X-inactivation, GMV, and GMCs. We examined variability in hearing thresholds in data from two large data sets containing data from more than 8500 normally hearing male and female listeners (Grant et al. (2021) and NHANES datasets [30, 31]). Variability in performance across males vs. across females was compared in each measure with the expectation of GMV (prediction 1). In addition, correlations between related measures (e.g., hearing thresholds at 500 and 1000 Hz) were examined within males vs. within females with the expectation that males would show higher correlations if differences in X-chromosome-activation patterns between males and females influence performance (GMCs—prediction 2). Finally, mean hearing thresholds were compared for males vs. females to determine if the better hearing sensitivity reported for females in previous studies was replicated in the current data.

## Methods

### Subjects, stimuli, and procedures

#### Grant et al. (2021) data set

Grant et al. [30] reported hearing thresholds for a large group of adult listeners. All subjects were active-duty members of the United States military and included listeners with no previous exposure to explosive blasts and with normal hearing, defined as having thresholds at or below 20 dB HL at audiometric test frequencies between 500 and 6000 Hz in both ears. A total of 1457 males (age range: 18–55 years, mean age: 25.8 years) and 486 females (age range 18–55 years; mean age: 28.2 years) fit these criteria and provided data for the analyses reported here. Audio stimuli were presented via headphones in an audiometric sound booth. Hearing thresholds were measured in each ear for pure tones at 500, 1000, 2000, 3000, 4000, and 6000 Hz. For both the Grant et al. and NHANES data sets, thresholds were determined based on the modified Hughson and Westlake presentation and scoring procedures recommended by Carhart and Jerger [32, 33]. These methods were approved as the recommended procedures for collection of hearing-threshold measures by the American Speech–Language–Hearing Association in 2005 [34].^2^

#### NHANES data set

The National Health and Nutrition Examination Survey (NHANES) is a research program conducted by the National Center for Health Statistics that provides publicly released data from interviews, physical examinations, and laboratory tests on adults and children in the United States [31]. The NHANES data set includes measures of hearing thresholds at 500, 1000, 2000, 3000, 4000, 6000, and 8000 Hz. NHANES data from eight annual surveys (1999, 2001, 2003, 2005, 2007, 2009, 2011, and 2015) were combined and examined to identify all listeners with normal hearing, defined as having thresholds at or below 20 dB HL at all test frequencies in both ears. A total of 7023 normal-hearing listeners were identified and their hearing thresholds were used in the analyses reported here (3133 males, age range: 12–67 years, mean age: 23.5 years; 3890 females, age range: 12–77, mean age: 26.7 years). Given that the hearing thresholds from the Grant et al. (2021) data set were exclusively from active-duty military personnel, it bears mention that the NHANES data specifically exclude this group of listeners. Details of test procedures for the NHANES hearing tests are at: https://wwwn.cdc.gov/Nchs/Nhanes/1999-2000/AUX1.htm and https://wwwn.cdc.gov/Nchs/Nhanes/1999-2000/AUX1.htm#Protocol_and_Procedure.

#### Statistical analyses

The hearing-threshold data were examined for evidence of GMV, GMCs, and sex differences in mean hearing thresholds. In the tests for potential GMV, variance ratios (male variance/female variance) were determined at each test frequency and Fisher’s variance-ratio test (*F* test) was used to determine whether the ratio at a given test frequency was significantly greater than 1.0 (consistent with GMV). In the tests for GMCs, Fisher’s *z*-test of differences between correlations was used to compare correlations between thresholds at different frequencies (or at the same frequency across ears) for males vs. the same correlations for females. The analyses testing for sex differences in mean thresholds compared thresholds as each test frequency using independent-samples *T* tests.

In each of these analyses, the data allowed multiple comparisons of male vs. female scores that were examined in separate tests. One concern with running these multiple planned comparisons is that even when the null hypothesis is true, it becomes likely that at least one test may show significant results based on the large number of tests. To address this issue, binomial tests were carried out to determine whether the number of significant results observed for a set of N comparisons was large enough to make it unlikely to have arisen based on chance alone. To clarify, consider the tests for GMV in the current data. A total of 26 separate comparisons were available in the current data allowing 26 tests for greater male variability. Assuming the null hypothesis is true (i.e., equal underlying variability in thresholds for males and females), running 26 tests with p set to 0.05 for each test has about a 70% probability of producing between one and three significant results (*P*(*x*) = (*n*!/((*n* − *x*)! (*x*!))) (*p*^*x*^) ((1 − *p*)^(*n* − *x*)^) with *n* = 26, *p* = 0.05, and *x* = 1, 2, or 3). The probability of four or more significant results is only about 4%. That is, four or more significant results across the 26 tests would be unlikely based on chance alone. The analyses reported below include binomial tests to determine if, given the number of tests run, the observed number of significant results was likely to occur based on chance alone.

## Results

**Fig. 3:**
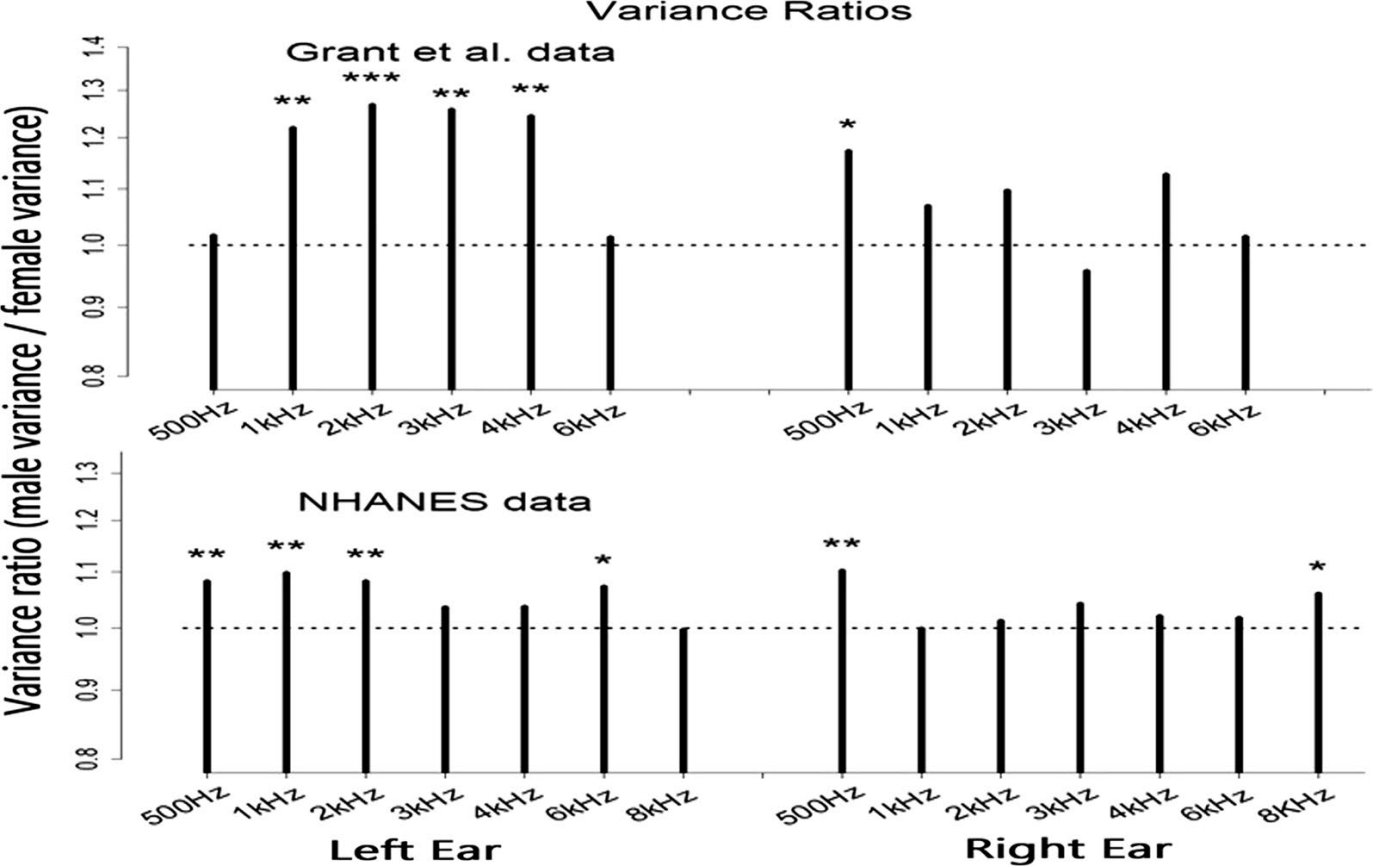
Variance ratios (male variance/female variance) for hearing thresholds at all test frequencies. Values are greater than 1.0 (dashed line) at frequencies showing GMV. Stars indicate significant differences from 1.0 (*p < 0.05; **p < 0.01; ***p < 0.001)

**Fig. 4:**
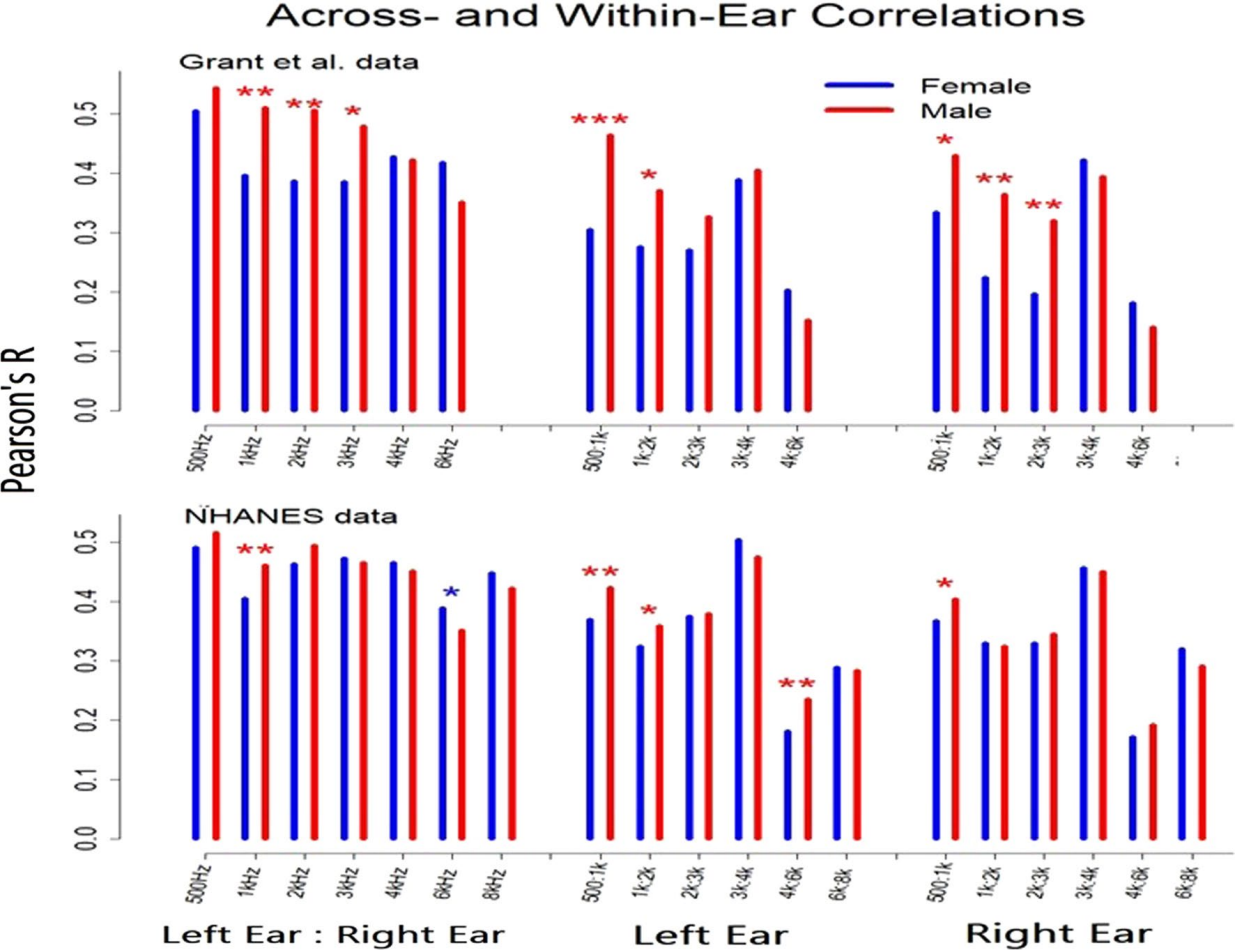
Correlations between hearing thresholds for males and females. Bars represent Pearson’s *R* correlations across ears at single test frequencies (left hand bars), between adjacent test frequencies in the left ear (middle bars) and between adjacent frequencies in the right ear (right-hand bars). Stars indicate significant differences in correlation for males vs. females (*p < 0.05; **p < 0.01, ***p < 0.001)

**Fig. 5:**
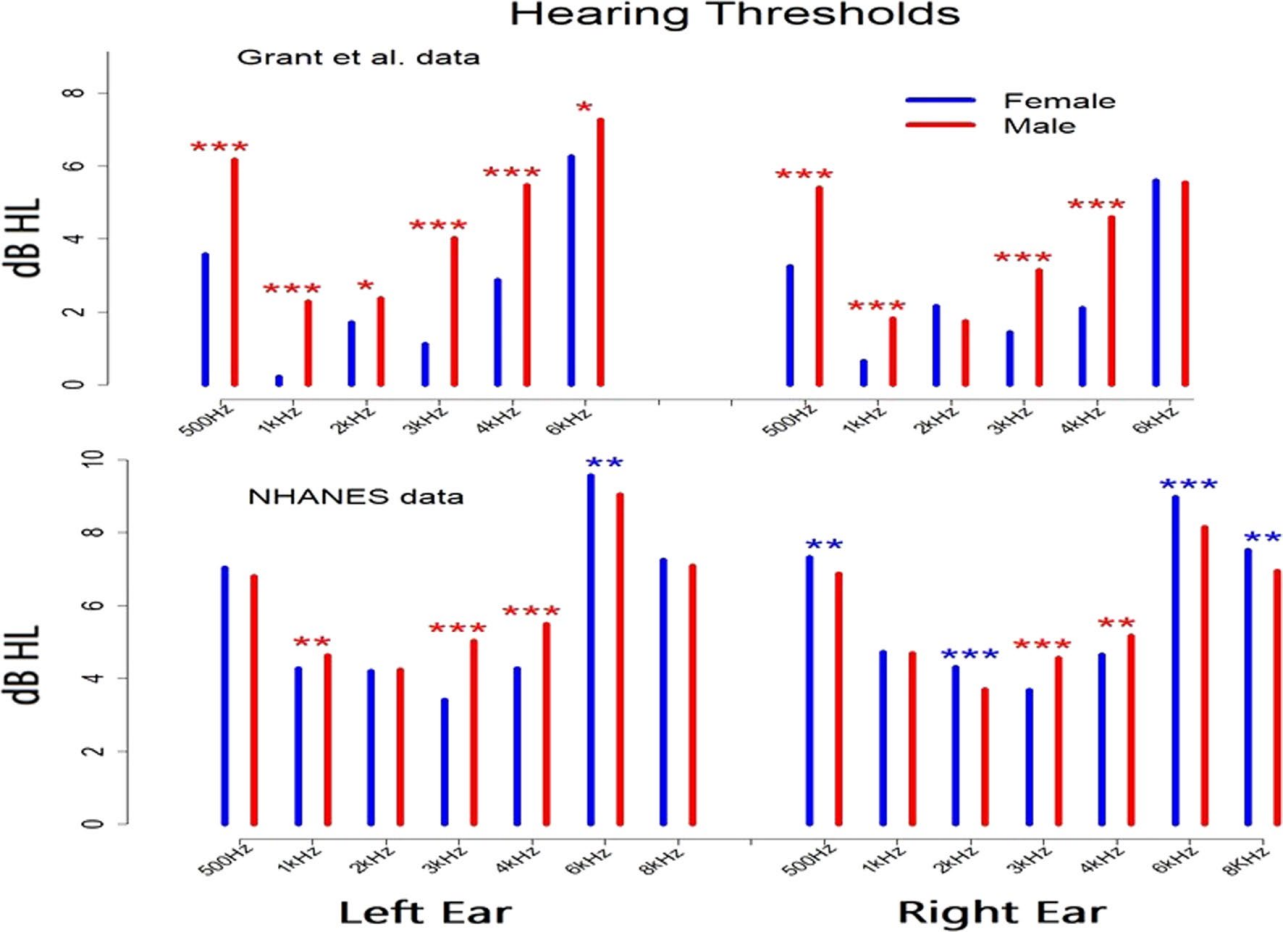
Hearing thresholds at all test frequencies for males and females. Stars indicate significant differences between mean thresholds for males vs. females (*p < 0.05; **p < 0.01, ***p < 0.001)

Figures 3, 4 and 5 present male:female differences on three different hearing measures: variability in hearing thresholds at each test frequency, correlations between both across-ear and between within-ear hearing thresholds, and mean thresholds at each frequency. The bars in each figure indicate the magnitude of male:female differences and *’s indicate statistically significant differences. The specific values represented by the bars in each figure and the probabilities that these male:female differences are due to chance are reported in Tables 1, 2 and 3.

**Table 1:** Variance ratios plotted in Fig. 3 and probabilities variance ratios = 1.0

Grant et al. data	500 Hz	1 kHz	2 kHz	3 kHz	4 kHz	6 kHz	
Left ear VarRatio	1.016	1.220	1.269	1.259	1.245	1.013	
prob VarRatio = 1.0	0.420	0.004	0.001	0.001	0.002	0.438	
Right ear VarRatio	1.173	1.068	1.096	0.957	1.127	1.014	
prob VarRatio = 1.0	0.018	0.192	0.112	0.270	0.057	0.430	

NHANES data	500 Hz	1 kHz	2 kHz	3 kHz	4 kHz	6 kHz	8 kHz
Left ear VarRatio	1.082	1.098	1.083	1.036	1.037	1.072	0.997
prob VarRatio = 1.0	0.010	0.003	0.010	0.151	0.145	0.020	0.464
Right Ear VarRatio	1.102	0.999	1.012	1.042	1.020	1.017	1.060
prob VarRatio = 1.0	0.002	0.492	0.361	0.114	0.282	0.306	0.044

**Table 2:** Pearson’s *R* correlations plotted in Fig. 4 and probabilities female *R* values = male *R* values

Grant et al. data	500 Hz	1 kHz	2 kHz	3 kHz	4 kHz	6 kHz	
Female across-ear Rs	0.504	0.396	0.386	0.386	0.428	0.418	
Male across-ear Rs	0.544	0.511	0.505	0.479	0.422	0.351	
prob (female *R* = male *R*)	0.151	0.003	0.002	0.015	0.450	0.067	

NHANES data	500 Hz	1 kHz	2 kHz	3 kHz	4 kHz	6 kHz	8 kHz
Female across-ear Rs	0.491	0.405	0.463	0.473	0.465	0.388	0.448
Male across-ear Rs	0.516	0.461	0.494	0.465	0.451	0.350	0.422
prob (female *R* = male *R*)	0.084	0.002	0.051	0.342	0.228	0.034	0.092

Grant et al. data	500:1k	1k:2k	2k:3k	3k:4k	4k:6k		
Female within-left ear Rs	0.305	0.276	0.270	0.388	0.203		
Male within-left ear Rs	0.464	0.370	0.326	0.405	0.152		
prob (female *R* = male *R*)	0.000	0.022	0.120	0.358	0.156		
Female within-right ear Rs	0.334	0.224	0.196	0.422	0.181		
Male within-right ear Rs	0.430	0.364	0.319	0.394	0.140		
prob (female *R* = male *R*)	0.017	0.002	0.006	0.261	0.213		

NHANES data	500:1k	1k:2k	2k:3k	3k:4k	4k:6k	6k:8k	
Female within-left ear Rs	0.369	0.324	0.374	0.504	0.181	0.288	
Male within-left ear Rs	0.423	0.359	0.379	0.475	0.235	0.284	
prob (female *R* = male *R*)	0.004	0.050	0.409	0.054	0.009	0.420	
Male within-right ear Rs	0.404	0.325	0.345	0.450	0.191	0.290	
Female within-right ear Rs	0.367	0.329	0.329	0.456	0.172	0.320	
prob (female *R* = male *R*)	0.036	0.412	0.230	0.370	0.201	0.087

**Table 3:** Mean hearing thresholds plotted in Fig. 5 and probabilities female mean thresholds = male mean thresholds

Grant et al. data	500 Hz	1 kHz	2 kHz	3 kHz	4 kHz	6 kHz	
Female left ear	3.570	0.216	1.708	1.121	2.881	6.255	
Male left ear	6.169	2.275	2.364	4.015	5.470	7.261	
prob (female mean = male mean)	0.000	0.000	0.026	0.000	0.000	0.010	
Female right ear	3.251	0.658	2.160	1.440	2.109	5.607	
Male right ear	5.408	1.809	1.750	3.140	4.595	5.539	
prob (female mean = male mean)	0.000	0.000	0.163	0.000	0.000	0.859	

NHANES data	500 Hz	1 kHz	2 kHz	3 kHz	4 kHz	6 kHz	8 kHz
Female left ear	7.027	4.265	4.194	3.404	4.279	9.555	7.243
Male left ear	6.805	4.638	4.240	5.019	5.488	9.033	7.081
prob (female mean = male mean)	0.107	0.005	0.760	0.000	0.000	0.002	0.368
Female right ear	7.319	4.719	4.303	3.685	4.647	8.963	7.517
Male right ear	6.866	4.684	3.695	4.566	5.171	8.139	6.939
prob (female mean = male mean)	0.001	0.798	0.000	0.000	0.001	0.000	0.001

### Variability in performance: males vs. females

Figure 3 shows variance ratios (male/female) for males and females at each test frequency in the two data sets with values from the Grant et al. (2021) data in the upper panel of the figure and values from the NHANES data in the lower panel. The specific values represented by the bars in Fig. 3 and the probabilities that these male:female differences are due to chance are reported in Table 1.  Males showed greater variance than females in both data sets. Variance ratios were greater than 1.0 (consistent with GMV) for 24 of 26 test frequencies across the two data sets with ratios significantly greater than 1.0 (*p* < 0.05) in 11 of these 26 tests and with *p* < 0.01 in eight cases [based on Fisher’s variance-ratio test (*F* test)]. The 26 bars in Fig. 3 represent 26 tests comparing male vs. female variances in thresholds at different frequencies. Given 26 tests, the probability of 11 or more tests showing p values below 0.05 is extremely low (*p* < 0.00000002).

### Correlations across measures: males vs. females

The panels of Fig. 4 show correlations between hearing thresholds collected from the two ears at the same frequency (far left in each panel) and between thresholds at adjacent test frequencies in the same-ear (middle and far right). The upper panel shows correlations for females and males in the Grant et al. (2021) data and the lower panel shows correlations for the NHANES data.The specific values represented by the bars in Fig. 4 and the probabilities that these male:female differences are due to chance are reported in Table 2.  Correlations were greater for males than females both across ears and within each ear. Across the two datasets, males exhibited higher correlations in 21 of 32 comparisons. Thirteen of those 21 differences were statistically significant (based on Fisher’s *z*-test of differences between correlations), 12 of which included a 500-, 1000-, 2000-, or 3000-Hz threshold. Females often showed higher correlations than males in correlations that did not involve these lower frequencies. However, only one of these differences was statistically significant (NHANES data, across-ear correlations at 6 kHz, *p* < 0.05).

Figure 4 shows 32 comparisons of correlations between hearing thresholds in males vs. females. Each of these planned comparisons was tested in a separate Fisher’s *Z* test. Assuming the null hypothesis is true and all male:female differences in the figure are due to chance, the probability of 13 or more of these tests showing p values less than 0.05 is less than 0.00000001.

### Differences in mean scores: males vs. females

Figure 5 shows mean thresholds for males and females at the frequencies tested in each data set. The specific values represented by the bars in Fig. 5 and the probabilities that these male:female differences are due to chance are reported in Table 3. In the Grant et al. (2021) data (upper panel of the figure), females show a clear advantage in hearing sensitivity across test frequencies and in both ears, with lower mean thresholds than males in 10 of the 12 pairwise comparisons with all 10 of these differences being statistically significant. This pattern replicates previous reports of lower absolute thresholds in females than males. The NHANES data did not show this female advantage in hearing sensitivity. For the 14 pairwise comparisons of female vs. male thresholds available in the NHANES data (lower panel of the figure), females showed lower thresholds in six cases and males showed lower thresholds in eight comparisons.

## Discussion

In the current data, hearing thresholds showed clear evidence of greater variability across males than across females and higher correlations across related measures within males than within females. The latter result supports the proposed link between GMV and the mosaic pattern of X-activation for females that is not present in males. Both GMV and GMCs were evident in both the Grant et al. and NHANES datasets.

The analyses comparing mean hearing thresholds for females vs. males showed different results in the two data sets. In the Grant et al. (2021) data, females showed lower hearing thresholds than males across a broad frequency range. In contrast, the NHANES data did not show any consistent female advantage in hearing thresholds, with males showing lower mean thresholds than females for about half of the frequencies tested (see Fig. 5). One difference between the two datasets is that subjects in the Grant et al. (2021) study had military experience while the NHANES subjects did not. If service in the military involves greater noise exposure for males than females, this could account for the male:female differences in threshold in the Grant et al. (2021) data which were not seen in the NHANES data. However, further examination of the two datasets did not appear to support this hypothesis. First, comparing thresholds for males in the two datasets showed slightly lower thresholds in the Grant et al. (2021) than the NHANES data. Obviously this would not be expected if increased noise exposure was specifically elevating thresholds for males in the Grant et al. (2021) data. Second, the differences between mean thresholds in the two datasets were even larger for females, again in the direction of lower thresholds in the Grant et al. (2021) data. So for both males and females, increased noise exposure associated with military service does not account for the pattern of threshold differences between the two datasets.

The higher thresholds for both males and females in the NHANEs dataset relative to the Grant et al. (2021) data and the more similar thresholds across males and females in the NHANES data may relate to differences in the testing environments. The Grant et al. (2021) data were collected in audiometric sound booths which were presumably very effective at eliminating ambient sound present outside the booths. The NHANES testing took place in the Mobile Examination Center which consists of connecting tractor trailers which do not contain sound booths. If a higher noise floor was present in the NHANES than the Grant et al. (2021) testing, this could account for the higher thresholds in the NHANES dataset and if absolute thresholds for females were in fact lower than for males, the higher noise floor might be expected to affect these lower thresholds more, making thresholds more similar for females and males in the NHANES dataset. The pattern of GMV in hearing thresholds in both datasets and the inconsistent pattern across the datasets in comparing means of females vs. males is another case where there does not appear to be a reliable association between sex-related differences in variability and in mean scores.

More sensitive hearing in females than males has been reported frequently [26–28], including in newborns [35, 36]. This female advantage in basic sensory/perceptual processing has been reported in other senses also, including color discrimination [37], olfaction [38], and taste [39, 40]. In cases where X-linked genes are involved, the mosaic pattern of X-activation for females may provide an advantage in terms of sensory processing. Visual color discrimination provides an interesting example that may apply to other sense data. Mutations on genes carried on the X chromosome can reduce red–green discrimination. These mutations make males much more vulnerable to red–green color-blindness than females because, for males, the mutated gene will be activated across all photoreceptor cells involved in red–green discrimination, but activated in only about 50% of those cells for females. For males, the mutation replaces photoreceptor cells allowing more accurate red–green discrimination with cells providing less accurate discrimination. However, for females, the mutation essentially adds additional, differently tuned processors without removing access to the originals. For females, the presence of this altered photoreceptor cell can produce added color sensitivity relative to males and to females who do not have an X-chromosome with this mutation [41–43]. This female advantage is analogous to benefiting from access to the “wisdom of crowds” as described by James Surowiecki in his book by that title [44]. Surowiecki describes “wise crowds” as having diversity and independence of opinion, which may characterize the mosaic X-activation pattern seen for females more than the uniform pattern of males. That is, if different alleles of a given X-linked gene (or genes) are associated with differences in basic sensory sensitivity, this may benefit females based on having two X chromosomes.

Across-ear correlations between hearing thresholds at a given frequency and within-ear correlations at adjacent frequencies were both higher for males than females. These GMCs are consistent with prediction 2, that for X-linked traits and characteristics, the mosaic pattern of X-activation in females should reduce correlations across related measures relative to correlations in males. Similar results involving click-evoked and spontaneous otoacoustic emissions (CEOAEs and SOAEs) were reported by McFadden et al. [45]. The number of SOAEs and the power of CEOAEs are greater in females than males and are linked to the better hearing sensitivity in females. McFadden and colleagues reported correlations for same-sex dizygotic twins on same-ear (right–right, left–left) and opposite-ear (right–left and left-ear) SOAEs and CEOAEs. In all eight comparisons made, male twins showed higher correlations than female twins. The authors viewed these GMCs as “unusual” and “anomalous” at the time. In a recent study, McFadden and colleagues reported that these CEOAE and SOAE measures show GMV along with the GMCs reported earlier [46]. GMCs have also been reported for a range of anatomical measures across brain regions in humans [8, 9, 21] and chimpanzees [6].

The majority of genes on the inactivated X chromosome are silenced in females and the resulting mosaic pattern of X-inactivation in females may contribute to GMV and GMCs for traits linked to these genes. It should be noted that about 15% of genes on this inactivated chromosome escape inactivation and are consistently expressed from both X chromosomes in human females. An additional 10–15% show variable escape and are expressed in some cell types and silenced in others.[47–49]. The current account of GMV and GMCs for traits linked to X-linked genes is most directly relevant for the 70–75% of the genes that are silenced on the inactivated X chromosome.

Sexual selection and sociocultural influences may each contribute to GMV. Sexual selection may play a particularly important role in “tournament species” where males can be seen as competitors in a tournament to mate with females, who are the judges in the tournament [1]. High variability on a given trait across males will place more males in the positive tail of the distribution for that trait, allowing them to meet the selection criterion set by a female. Sociocultural factors may also contribute to GMV to the extent that females receive less opportunity and encouragement to develop abilities placing them in the high-performance tails of distributions for some skills [50].

Although sexual selection and sociocultural factors may contribute to GMV for certain traits, a wide variety of traits and morphological measures show GMV with no apparent link to either selection or societal factors (e.g., the GMV in the hearing thresholds reported above, in additional hearing measures [46], in birthweights, and in blood parameter measures [4]). The difference in the pattern of X-activation between males and females represented in Fig. 1 predates current mammalian species by more than 100 million years [13]. Therefore, to the extent that these differences produce GMV, this greater variability in males may be independent of selection and sociocultural pressures influencing a specific species.

A final point relates to whether GMV in various traits is common across the animal kingdom or is limited to certain animal groups. The factors contributing to GMV that are the focus of this paper (i.e., heterogametic sex chromosomes and uniform X-chromosome activation in males vs. homogametic with mosaic X-inactivation in females) are present in placental mammals but are not shared by some other groups. For example, for birds and butterflies, females are heterogametic (ZW) while males are homogametic (ZZ). In a study examining variation in body size in taxa differing in which sex is heterogametic, greater variability was consistently present for the heterogametic sex (females for bird and butterflies, males for mammals and other insects) [7]. Assuming this result characterizes other traits, the GMV seen for many traits in placental mammals may not hold for species where females are the heterogametic sex.

In 2016, NIH mandated the inclusion of sex as a biological variable (SABV) in biomedical research, to include not only enrolling males and females in similar numbers, but that: “sex as a biological factor will be factored into research designs, analyses, and reporting in vertebrate animal and human studies” [51]. However, a recent meta-analysis of over 3000 articles in prominent Neuroscience and Psychiatry journals indicated that the majority of papers did not analyze by sex ([52], see also [53]). In addition, studies that have included sex as a variable in research designs and analyses have almost exclusively focused on mean differences between males and females and not examined possible differences in variability.

As this paper is being submitted, there is a worldwide pandemic involving coronavirus disease 19 (Covid 19). Like many other infectious diseases (including ones involving other types of coronavirus), males are at greater risk of severe outcomes including mortality from contracting Covid 19 [54–56]. These sex differences are associated with X-linked genetic differences [57–59]. Along with mosaic activation of X-linked genes providing potential benefit to immunological response, a second mechanism increasing female immune response may be the escape from inactivation present in 25–30% of X-linked genes in inactivated X chromosomes for females [47–49]. Escape from inactivation may provide a “double dose” of immunological benefit relative to males for genes that provide this benefit. The studies reporting male–female differences in vulnerability to infectious disease do not appear to include any that explicitly tested for sex differences in variability in their dependent measures.

## Perspectives and significance

Greater variability across males than females over a wide range of phenotypic traits is well-documented for many species. Here, we report hearing-threshold data for > 8500 human listeners with normal hearing that show this greater male variability. The data analyses support a link between this greater variability in males and consistent vs. mosaic patterns of X-chromosome activation in males vs. females. This male:female difference predates current species by over 100 million years and thus predates selection pressures on current species that may also contribute to greater male variability. A clearer understanding of how, and to what extent, sex differences in X-chromosome activation patterns contribute to the greater male variability seen in many human characteristics will improve our understanding of sex differences in vulnerability to disease and in a range of additional phenotypic traits and anatomic characteristics.

## Supplementary Information

**Additional file 1.** Individual hearing thresholds - Grant et al & NHANES datasets.

## Data Availability

All data analyzed in this study are included in this article and in the file listed below under Supplementary Information (Additional file 1).

## Footnotes

1 Differences in the degree of imbalance of X-chromosome inactivation can produce phenotypic differences between monozygotic (MZ) female twins (“identical” twins). More skewed X-inactivation patterns for one of two female MZ twins have been linked to deficits in red–green color vision and vulnerabilities to Duchenne muscular dystrophy and hemophilia [60–63]. These differences would not occur between male MZ twins given the identical patterns of X-chromosome activation for both twins. Male MZ twins are, in this way, more identical than female MZ twins.

2 The methods involve an ascending-presentation-level technique. Thresholds were determined for pure tones of 1 to 2 s duration. Intervals between successive tones were varied, but were no shorter than the test tone. The level for the first presentation for each test tone was set below the expected threshold. The level for each succeeding presentation was based on the previous response. After each failure to respond to a test signal the level was increased in 5-dB steps until a detection response occurred. After this response, presentation level was decreased by 10 dB and another ascending series began. Hearing thresholds were defined as the lowest dB level at which responses occurred in at least half of a series of ascending trials at a single level. The minimum number of responses required to determine a threshold was two responses out of three presentations at a single level.
